# Post-stimulatory activity in primate auditory cortex evoked by sensory stimulation during passive listening

**DOI:** 10.1038/s41598-020-70397-0

**Published:** 2020-08-17

**Authors:** James E. Cooke, Julie J. Lee, Edward L. Bartlett, Xiaoqin Wang, Daniel Bendor

**Affiliations:** 1 Institute of Behavioural Neuroscience (IBN), University College London (UCL), London, WC1H 0AP UK; 2 Institute of Ophthalmology, University College London (UCL), London, WC1H 0AP UK; 3 Departments of Biological Sciences and Biomedical Engineering, Purdue University, West Lafayette, 47907 USA; 4 Departments of Biomedical Engineering, Johns Hopkins University, Baltimore, 21205 USA

**Keywords:** Neuroscience, Auditory system, Sensory processing

## Abstract

Under certain circumstances, cortical neurons are capable of elevating their firing for long durations in the absence of a stimulus. Such activity has typically been observed and interpreted in the context of performance of a behavioural task. Here we investigated whether post-stimulatory activity is observed in auditory cortex and the medial geniculate body of the thalamus in the absence of any explicit behavioural task. We recorded spiking activity from single units in the auditory cortex (fields A1, R and RT) and auditory thalamus of awake, passively-listening marmosets. We observed post-stimulatory activity that lasted for hundreds of milliseconds following the termination of the acoustic stimulus. Post-stimulatory activity was observed following both adapting, sustained and suppressed response profiles during the stimulus. These response types were observed across all cortical fields tested, but were largely absent from the auditory thalamus. As well as being of shorter duration, thalamic post-stimulatory activity emerged following a longer latency than in cortex, indicating that post-stimulatory activity may be generated within auditory cortex during passive listening. Given that these responses were observed in the absence of an explicit behavioural task, post-stimulatory activity in sensory cortex may play a functional role in processes such as echoic memory and temporal integration that occur during passive listening.

## Introduction

Neurons in a number of cortical areas have been found to fire for durations on the order of seconds in the absence of sensory stimulation^1–7^. This phenomenon was first described in the context of working memory tasks^8^. In such tasks, subjects are typically presented with a cue and are required to respond after a delay period, during which the cue is no longer available. Neurons in the dorsolateral prefrontal cortex of macaque have been found to fire continuously during such delay periods, providing a possible substrate for the maintenance of information in working memory^1,2,6,9–14^. In keeping with this proposed function, suppressing this activity has been shown to impair performance on working memory tasks^15–17^.

Such activity has been found to carry spatial information such as the location of visual stimulus^1,3–5^ as well as the direction of a forthcoming saccade^6^. Persistent activity has been reported in multiple cortical areas with differing functional properties. The intrinsic timescales of neural activity have been found to vary between cortical regions^18^ in a manner that predicts the functional properties of persistent activity across cortical regions^19,20^. “In addition to prefrontal activity, sensory areas have been found to encode stimulus information across a delay period in the form of post-stimulatory activity. In a tone discrimination task where the tones were separated by a one-second delay period, neurons in the auditory cortex of the macaque were found to elevate their firing during the delay period^7^. The presence of persistent delay period activity in auditory cortex during working memory tasks has been widely observed in the macaque^22–25^. Such activity has also been observed in rodent auditory cortex during working memory tasks and reference memory tasks^26^ as well as during auditory-signaled response preparation^27^ and following delay conditioning^28,29^. In addition to carrying stimulus information, persistent activity in auditory cortex has been found to relate to reinforcers and behavioral responses^30–32^. Post-stimulatory activity has also been reported in human auditory cortex, as measured by magnetoencephalography (MEG)^21,33^. These findings demonstrate the ability of auditory cortical neurons to maintain their firing for hundreds of milliseconds in the absence of a coincident stimulus.”

During passive listening, the termination of an auditory stimulus routinely evokes transient offset responses in auditory cortical neurons, typically lasting up to tens of milliseconds^34–37^. In the cat, the tonal receptive fields of offset responses are often similar to the tuning of responses evoked at the onset of the stimulus although onset and offset receptive fields can also vary their tuning properties^36^. In the mouse however, the tuning of onset and offset responses is largely distinct^35^. Despite this lack of consistency in receptive field dynamics, the ability of auditory cortical networks to routinely generate activity following the termination of an effective auditory stimulus is a consistent feature across species. The maximum duration of activity that can be evoked following stimulus offset, however, is currently unknown.

Here, we investigated whether it is possible to evoke post-stimulatory activity in auditory cortical neurons in the absence of a behavioural task and aimed to characterise the properties of such activity. We analysed activity from single units in the auditory cortex (fields A1, R and RT) and thalamus of awake, passively-listening marmosets that were presented an array of auditory stimuli during experiments not concerned with investigating post-stimulatory activity^38–41^. We observed post-stimulatory activity lasting for hundreds of milliseconds following the termination of the acoustic stimulus in a sub-population of auditory cortical neurons. This activity followed a variety of response profiles during sensory stimulation, including adapting, sustained and suppressed responses. Post-stimulus activity had a shorter latency and was of longer duration in cortex than in thalamus, indicating that the mechanisms underlying this activity may be primarily cortical.

## Results

### Post-stimulatory activity is observed in auditory cortex units during passive listening

**Figure 1:**
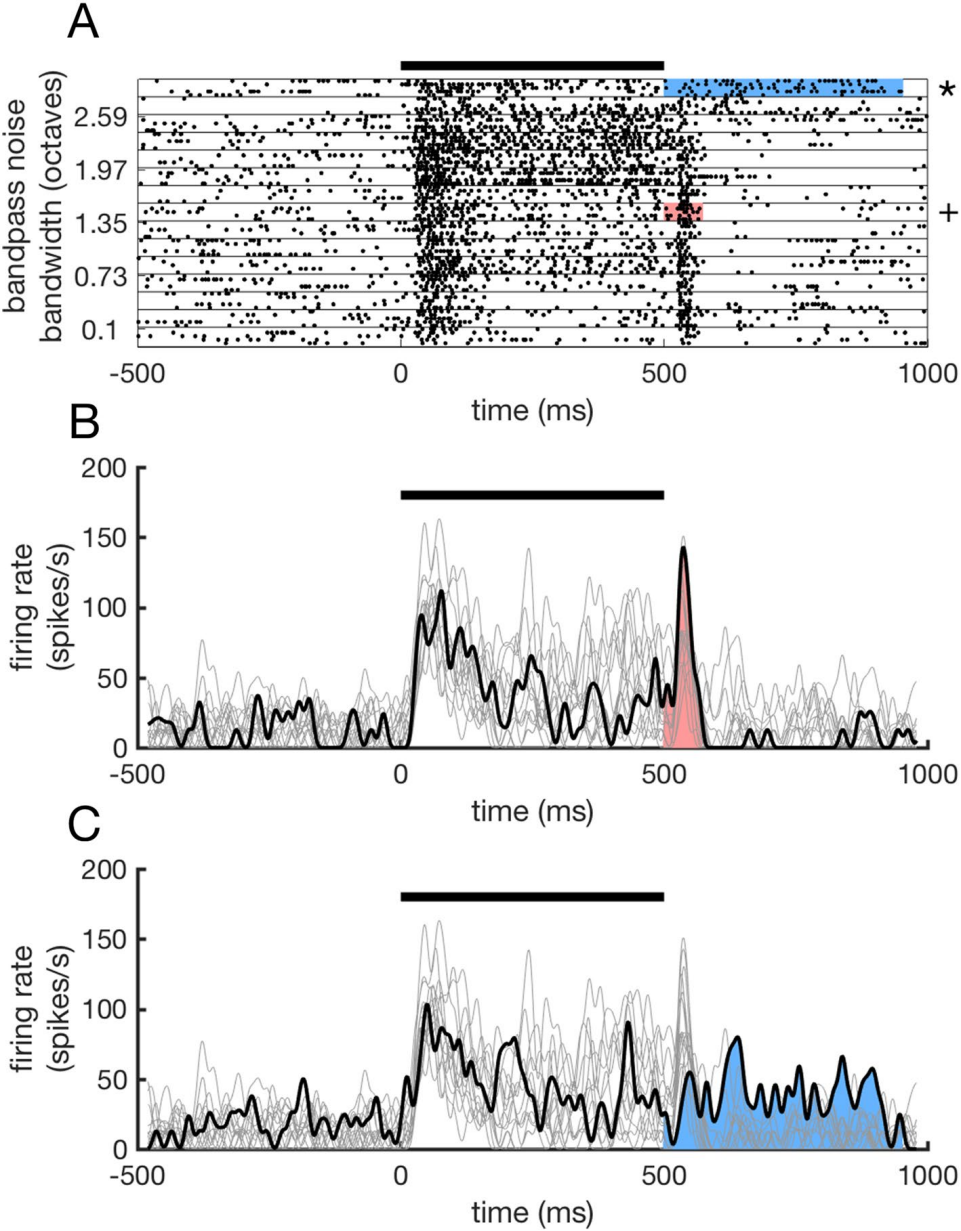
Post-stimulatory activity during passive listening. (**A**) Example unit presented with bandpass noise stimuli of varying bandwidth. This units displays short-duration offset responses to the majority of stimuli presented but also post-stimulatory activity in response to particular bandwidths. Spike rasters showing a representative offset response are indicated by the +. Spike rasters showing post-stimulatory activity are indicated by the *. The within-stimulus period is indicated by the black bar while the duration of significant post-stimulus activity on these trials in indicated by grey shading. (**B**) The black trace corresponds to the PSTH of spiking activity indicated by the + symbol in A while the grey traces are the PSTHs associated with all other stimuli. The black bar indicates the stimulus duration. The coloured area of the PSTH indicates the duration of the post-stimulus response that was elevated above baseline. (**C**) PSTH of spiking activity indicated by the * symbol in (**A**), showing post-stimulatory activity in the same unit.

The data analysed in this report were based on a database of 1557 single units recorded from the auditory cortex of 4 passively listening marmosets during the presentation of a variety of auditory stimuli in previous experiments. The stimuli presented were unmodulated pure tones, amplitude-modulated tones, white noise, band-pass noise and click trains. Of these units, 1,188 met our criteria to be included in further analysis (see “Methods”). Units were required to show an elevation in firing > 2 standard deviations over mean baseline firing on at least half of the trials that was longer in duration than for baseline activity observed during the pre-stimulus period. For the initial analysis of post-stimulatory activity units were not separated by the cortical field they were recorded from. Post-stimulatory activity was observed in a subset of these single units in response to particular stimuli (Fig. 1A). Short-duration offset responses were commonly observed, with 77.78% (N = 924/1,188) of units showing significant post-stimulus activity beginning within 50 ms of stimulus offset (Fig. 1B). By searching the stimulus space, it was possible to evoke post-stimulatory activity in 39.31% (N = 467/1,188) of the units recorded (see “Methods”) (Fig. 1C).

**Figure 2:**
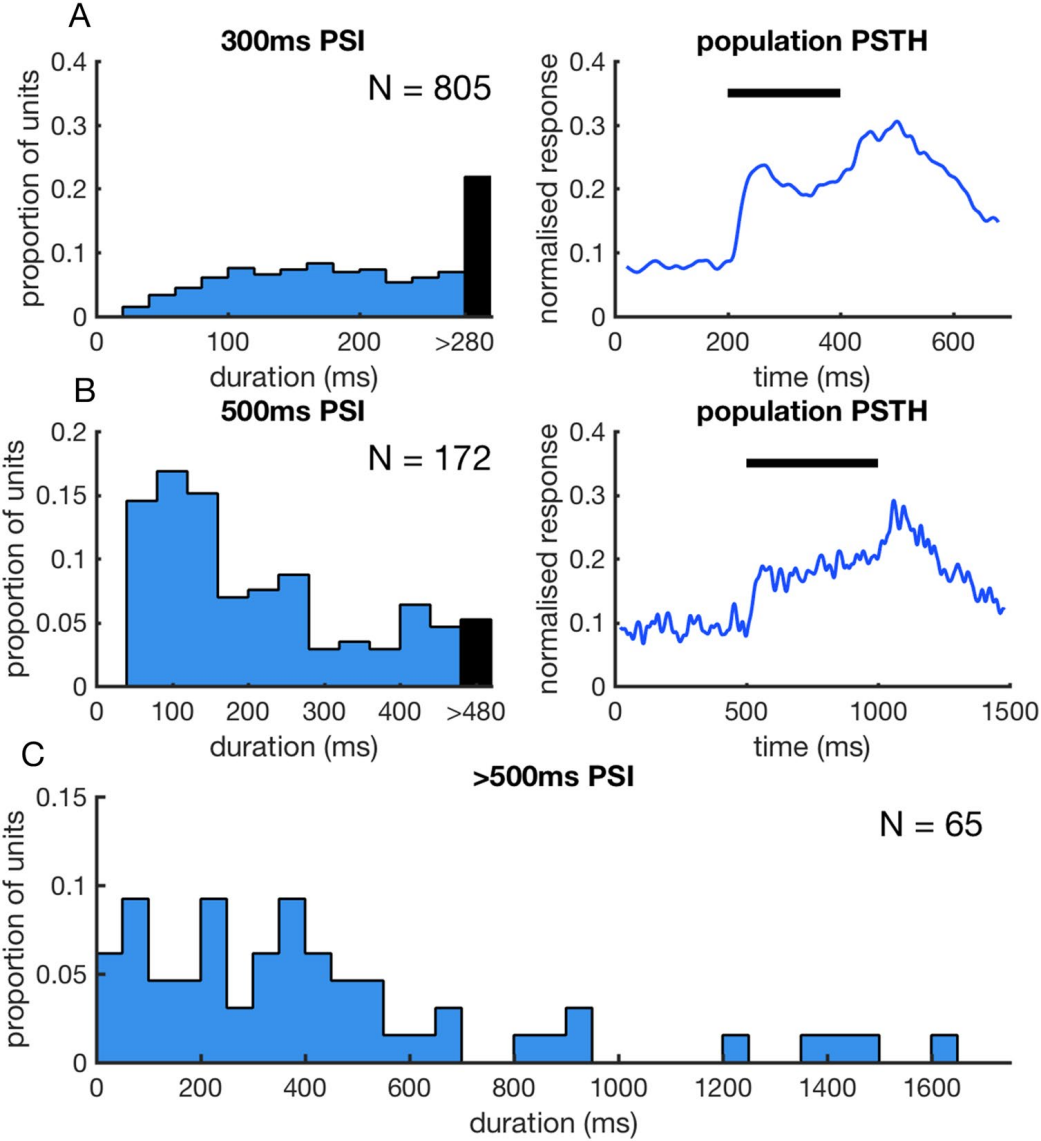
Duration of post-stimulus activity. (**A**) Left panel: Distribution of longest duration post-stimulus activity for 805 single units presented with 200 ms tone and noise stimuli followed by a 300 ms post-stimulus interval (PSI). The black bar indicates units that showed post-stimulatory activity lasting for the entire PSI. Proportion of units here and throughout the paper refers to the fraction of the total number of units included in the particular analysis. Right panel: Normalised population PSTH for these responses. (**B**) Left panel: Distribution of longest duration post-stimulus activity for 172 single units presented with 500 ms tone and noise stimuli followed by a 500 ms PSI. Right panel: Normalised population PSTH for these responses. (**C**) Distribution of longest duration post-stimulus activity for 65 single units presented with tone, noise and click stimuli that were followed by a PSI > 500 ms.

Stimulus parameters such as stimulus type and post-stimulus interval were varied across the neurons recorded. In order to quantify the duration of the observed post-stimulus activity, the analysis was initially restricted to stimuli with a post-stimulus interval (PSI) of 300 ms. This subset consisted of responses to tone and noise stimuli lasting 200 ms. For the population of 805 units recorded with these stimulus parameters, the longest duration post-stimulus response observed for each unit exceeded this 300 ms interval in 21.86% of units (Fig. 2A). When the PSI was extended to 500 ms for tone and noise stimuli 5.23% of the 172 units tested exceeded this 500 ms interval (Fig. 2B). For units presented with tone, noise and click stimuli followed by a PSI of > 500 ms (N = 65), the range of post-stimulus response durations spanned from 12 to 1681 ms (Fig. 2C).

**Figure 3:**
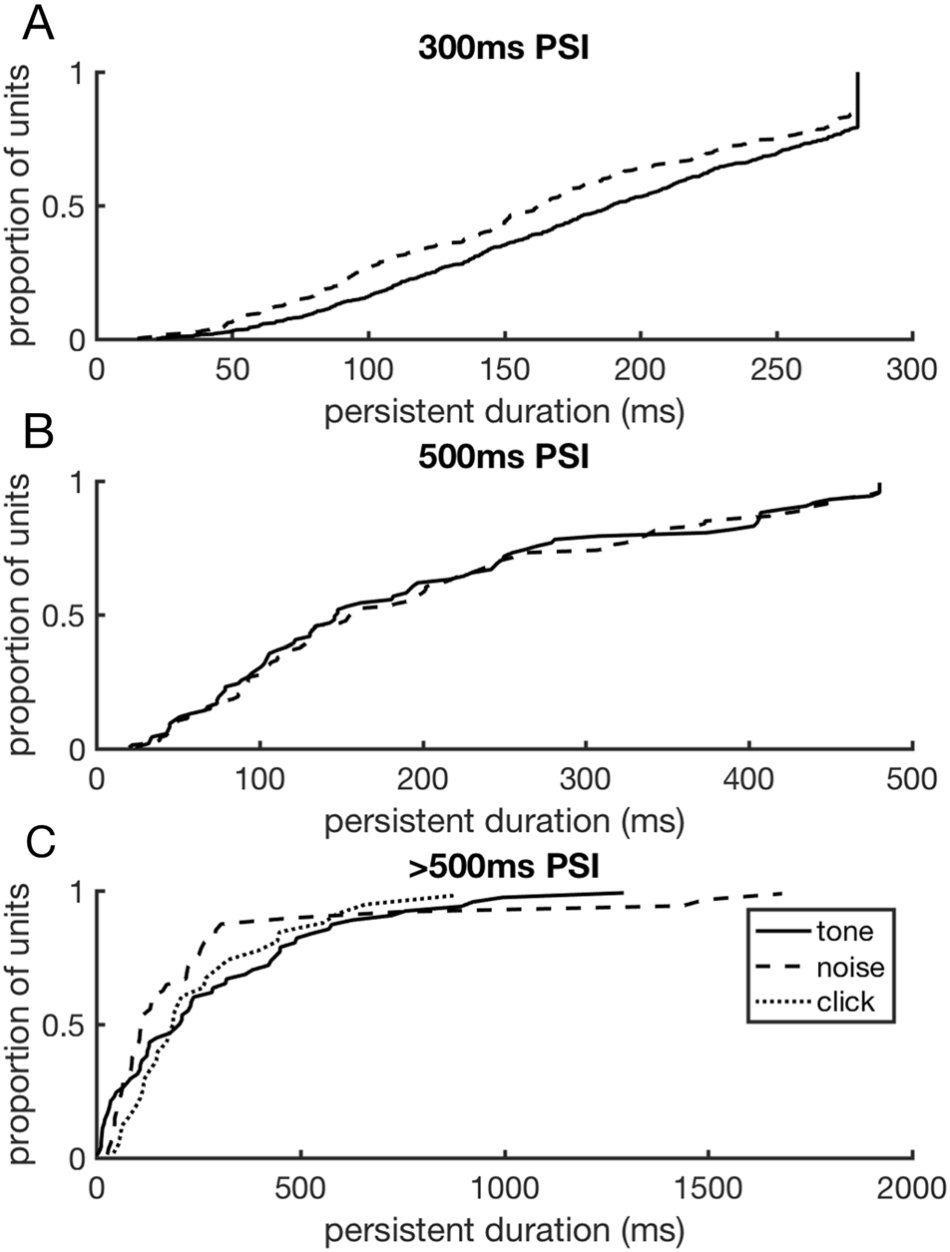
Distribution of post-stimulatory activity durations produced by different stimulus types. (**A**) Comparison of the distributions of post-stimulatory activity duration produced by tone and noise stimuli followed by a 300 ms PSI. Post-stimulus activity in response to tone stimuli was found to be of longer duration than that produced by noise stimuli when tested with this PSI. (**B**) Comparison of post-stimulatory activity duration produced by tone and noise stimuli followed by a 500 ms PSI. No significant difference in the means of these distributions were observed. (**C**) Comparison of post-stimulatory activity duration produced by tone, noise and click stimuli followed by a 500 ms PSI. No significant difference in the means of these distributions were observed.

Post-stimulatory activity was observed for all stimulus types tested. For stimuli with a PSI of 300 ms, tone stimuli produced significantly longer duration post-stimulatory activity (N units = 694, mean = 186.93 ms) than noise stimuli (N units = 308, mean = 167.96 ms; Two-Sample t-test, p < 0.001) (Fig. 3A). For stimuli with a PSI of 500 ms, no significant difference was found between the duration of post-stimulus activity in response to tone (N units = 80, mean = 196.3 ms) and noise stimuli (N units = 110, mean = 201.49 ms) (Two-Sample t-test, p = 0.8) (Fig. 3B). For stimuli with a PSI of > 500 ms, neurons were tested with tone (N units = 59, mean = 280.93 ms), noise (N units = 44, mean = 246.05 ms) and click train (N units = 29, mean = 256.21 ms) stimuli. No significant difference was observed in the duration of post-stimulus activity produced by these different stimulus types (ANOVA(2); p = 0.84, Fig. 3C).

### Post-stimulatory activity is observed following adapting, sustained and suppressed responses

**Figure 4:**
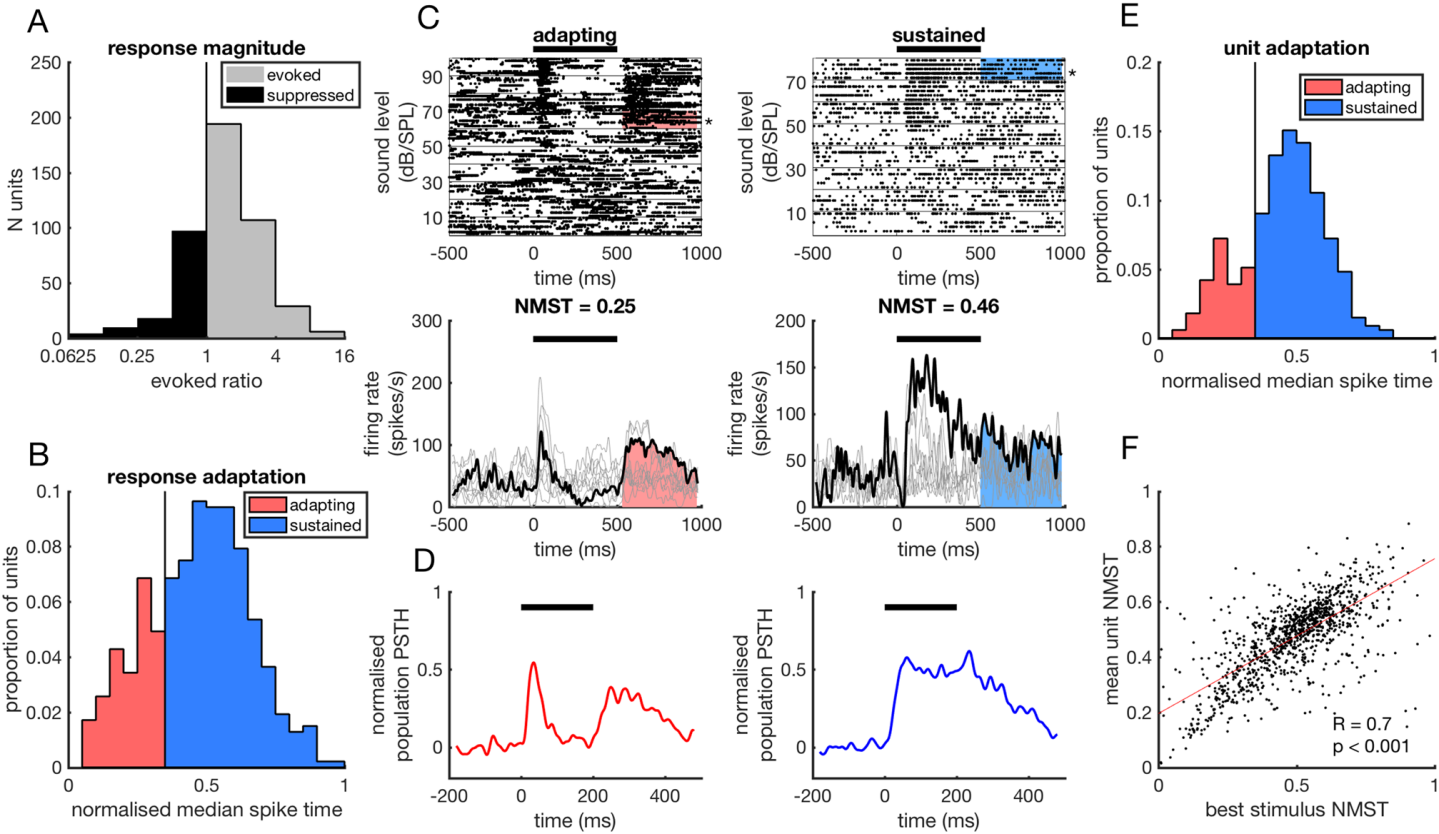
Post-stimulus activity following adapting and sustained responses. (**A**) Distribution of evoked ratios (N = 467). The vertical line at 1 indicates the threshold separating evoked (N units = 332) from suppressed responses (N units = 135). (**B**) Distribution of normalised median spike times of responses to the stimulus that evoked post-stimulatory activity (N units = 332). The vertical line at 0.35 indicates the threshold separating adapting (N units = 78) from sustained responses (N units = 254). (**C**) Upper panels: Raster plots for two units, both presented with 500 ms pure tone stimuli at their best frequency with varying sound levels. The left panel shows an adapting response while the right panel shows a sustained response. Lower panels: Corresponding PSTHs for the two units shown in upper panels. The PSTH in black is the response to the stimulus that produced the longest post-stimulatory activity for that unit. Titles above PSTHs show the normalised median spike time for this response. (**D**) Normalised population PSTH for adapting (left panel) and sustained (right panel) responses. (**E**) Distribution of normalised median spike times calculated from the mean responses across all stimuli for each unit (N units = 332, adapting N = 76, sustained N = 256). (**F**) Scatter plot between unit and response normalised median spike times showing a positive correlation.

The response profiles observed within auditory stimulation were examined next in order to gain insight into the dynamics that might produce this post-stimulatory activity. For the units that showed post-stimulatory activity (N = 467), within-stimulus evoked activity was quantified by taking the ratio of the within-stimulus firing rate and the baseline firing rate preceding sensory stimulation to produce an evoked ratio (Fig. 4A). An evoked ratio > 1 would indicate that the within-stimulus firing rate was increased with respect to baseline firing while values < 1 would indicate suppression of firing in the within-stimulus period. The dynamics of responses with an evoked ratio of > 1 were first analysed. For each response in this group the median within-stimulus spike time was calculated as a measure of the extent of adaptation that occurred during sensory stimulation. This median spike time was normalised by the duration of the stimulus, producing a normalised median spike time (NMST) for each unit between 1 and 0. This measure of adaptation results in fast adapting responses being associated with values near to zero and ramping responses being associated with values near one. For units with an evoked ratio of > 1 (N units = 332 of 467) the majority of units had NMSTs in the middle of this range, indicating a prevalence of sustained response profiles (Fig. 4B). In order to separate the overlapping adapting and sustained populations, adapting responses were defined as those with an NMST of < 0.35 (N units = 78 of 332) while sustained responses were defined as those with an NMST of > 0.35 (N units = 254 of 332, Fig. 4B). Ramping responses with NMSTs near 1 were rare and were classed as sustained responses here. Post-stimulatory activity was observed following both adapting and sustained responses (Fig. 4C,D). NMSTs were also calculated for the mean response to all stimuli for a single unit and showed a qualitatively similar distribution to the response NMSTs that were calculated for the stimulus that produced the longest duration post-stimulatory activity (N units = 332, adapting N = 76, sustained N = 256, Fig. 4E). These two measures also showed a significant positive correlation (r = 0.7, p < 0.001) indicating that within-stimulus response profiles that precede post-stimulatory activity for a single effective stimulus are representative of the average response dynamics of the units (Fig. 4F).

**Figure 5:**
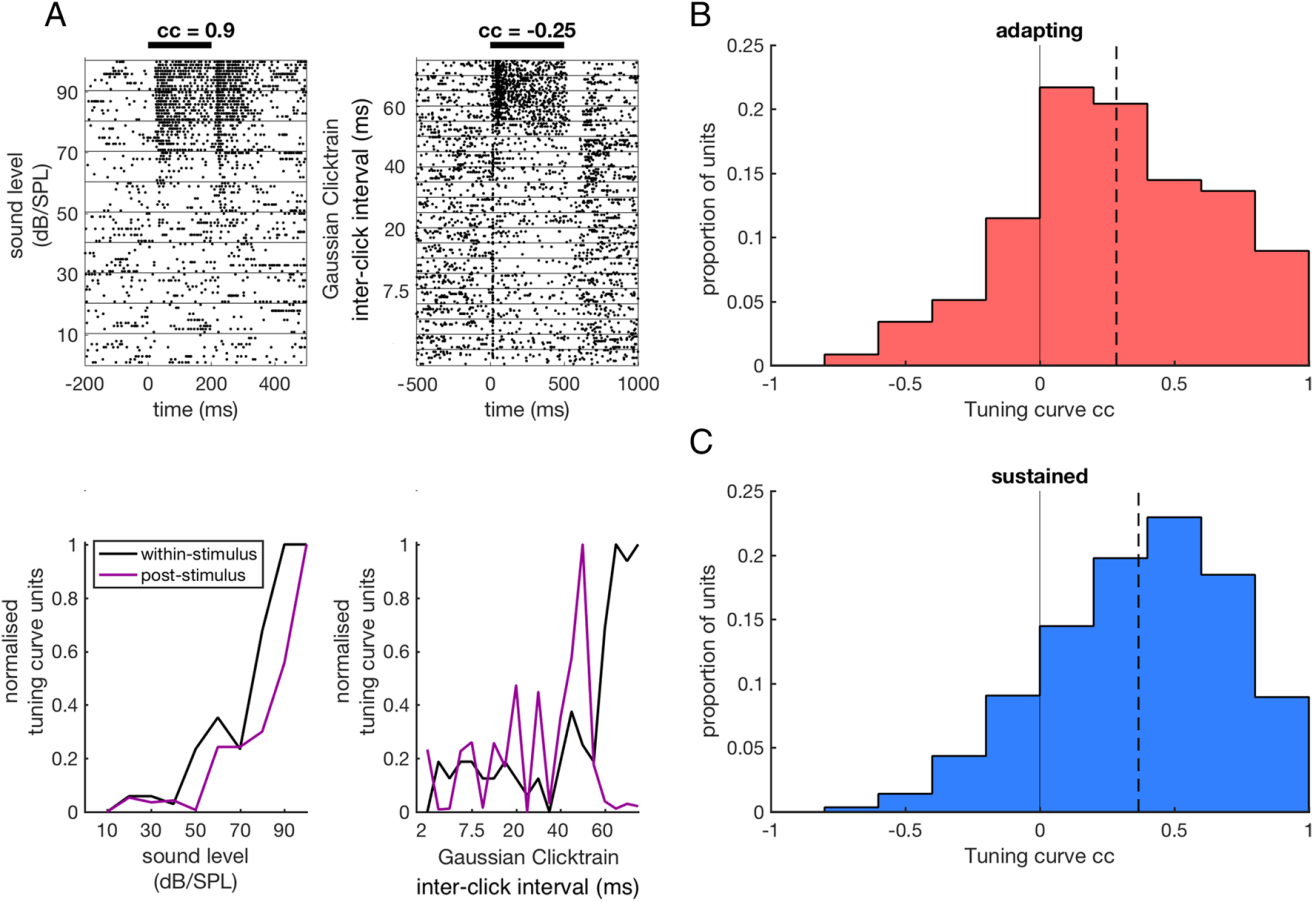
Within and post-stimulus tuning curves. (**A**). Rasterplots of responses (upper panels) and tuning curves (lower panels) for two units. The left panel shows the responses of a unit presented with 200 ms pure tone stimuli of varying sound level presented at the neuron’s best frequency. The right panel shows the responses of a unit presented with click train stimuli lasting 500 ms with varying click rates. Black curves show the within-stimulus tuning curves, calculated by taking the normalised peak firing rate in response to each stimulus. The purple curves show the post-stimulus tuning curve for the unit, calculated by measuring the duration of significantly elevated firing in the post-stimulus interval. (**B**) The distribution of correlation coefficients (ccs) between the within and post-stimulus tuning curves for units showing adapting responses to their best stimulus. The broken line indicates the median cc for the distribution. (**C**) Distribution of ccs between the within and post-stimulus tuning curves for units showing sustained responses to their best stimulus.

The relationship between within-stimulus response magnitude and the duration of post-stimulus activity was next examined. Within-stimulus tuning curves were calculated by measuring the peak firing rate during the stimulus time period in the mean PSTH for each stimulus (Fig. 5A). Post-stimulus tuning curves were calculated by measuring the duration of significant post-stimulus activity produced by each stimulus. A correlation coefficient (cc) was then calculated for these tuning curve pairs for each unit by correlating the peak firing rates across stimuli during the within period with the duration of post-stimulus activity. Tuning curves showed a significant positive correlation across the population of units classified as having adapting responses to their best stimulus (N units = 78, mean = 0.28, t-test; p < 0.001) (Fig. 5B). This was also the case for units that showed sustained responses to their best stimulus (N units = 254, mean = 0.37, t-test; p < 0.001) (Fig. 5C). Tuning curves for units that showed a sustained response (Fig. 5C) showed a significantly greater correlation across the population than units that showed an adapting response to their best stimulus (Fig. 5B) (Adapting mean cc = 0.28, Sustained mean cc = 0.37, two sample t-test; p = 0.001).

**Figure 6:**
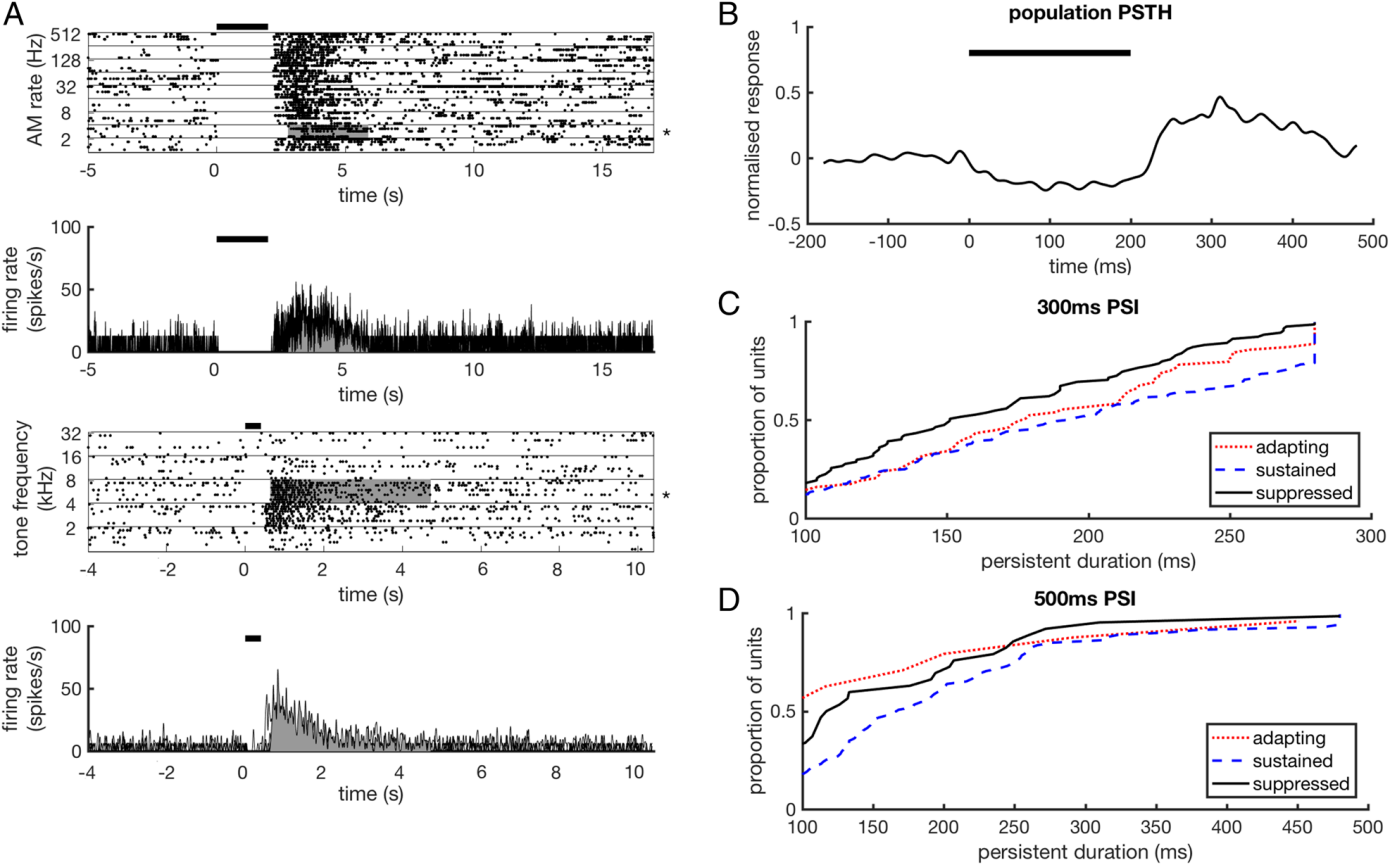
Post-stimulus activity following stimulus induced suppression. (**A**) Raster plots and corresponding PSTHs for two suppressed units. The responses in the upper two panels are from a neuron presented with 2 s amplitude modulated (AM) pure tones presented at the neuron’s best frequency, with the rate of amplitude modulation varied. The responses in the lower two panels are from a neuron presented with 200 ms pure tone stimuli of varying frequency. PSTHs in bold indicate the responses to the stimulus that produced the longest duration post-stimulus activity for that unit. (**B**) Normalised population PSTH for units showing within-stimulus period suppression when presented with noise, tone or click stimuli lasting 200 ms. The stimulus that evoked the longest duration post-stimulus activity was used for each unit, resulting in a variety of stimuli contributing to the population PSTH. (**C**) Cumulative distribution of longest duration post-stimulus activity for adapting, sustained and suppressed responses for stimuli with a post-stimulus interval of 300 ms. (**D**) Cumulative distribution of post-stimulus response durations for stimuli with a post-stimulus interval of 500 ms.

Not all units that showed significant post-stimulus activity showed evoked within-stimulus auditory activity in the within-stimulus interval. These units, associated with an evoked ratio of < 1, were examined next (N units 135 of 467). Units in this population typically demonstrated within-stimulus suppression (Fig. 6A,B). Elevated post-stimulus activity can therefore be observed following adapting, sustained and suppressed within-stimulus response profiles. For stimuli with a PSI of 300 ms, significant variation in post-stimulus response duration was observed between adapting (N units = 66, mean = 181.24 ms), sustained (N units = 167, mean = 190.98 ms) and suppressed (N units = 96, mean = 161.3 ms) response subtypes (Fig. 6C) (ANOVA(2); p = 0.003). Pairwise significance tests between group means (Tukey–Kramer test, corrected for multiple comparisons) indicated that sustained responses were followed by longer duration post-stimulus activity (mean = 190.98) than suppressed responses (mean = 161.3) but was not significantly different from adapting responses (mean = 181.24) (adapting vs sustained: p = 0.58, adapting vs suppressed: p = 0.16, sustained vs suppressed: p = 0.002). For stimuli with a PSI of 500 ms, no significant differences were observed between the durations of post-stimulus activity for adapting (N units = 12, mean = 136.67 ms), sustained (N units = 76, mean = 194.99 ms) and suppressed (N units = 31, mean = 153.97 ms) response types (ANOVA(2); p = 0.08) (Fig. 6D).

A significant positive correlation was observed between baseline firing rates and the duration of post-stimulus activity across adapting (N units = 78, r = 0.32, p < 0.001), sustained (N units = 254, r = 0.23, p < 0.001) and suppressed units (N units = 135, r = 0.1, p < 0.01). We quantified the burstiness of baseline firing using the coefficient of variation, *CV*:

$${CV = \frac{\sigma }{\mu }}$$

where the standard deviation of the inter-spike-intervals is *σ* and the mean inter-spike-interval is *μ*. A significant positive correlation was also observed between the coefficient of variation and the duration of post-stimulus activity across adapting (N units = 78, r = 0.23, p < 0.001), sustained (N units = 254, r = 0.22, p < 0.001) and suppressed units (N units = 135, r = 0.22, p < 0.001).

### Post-stimulus activity emerges in cortex

**Figure 7:**
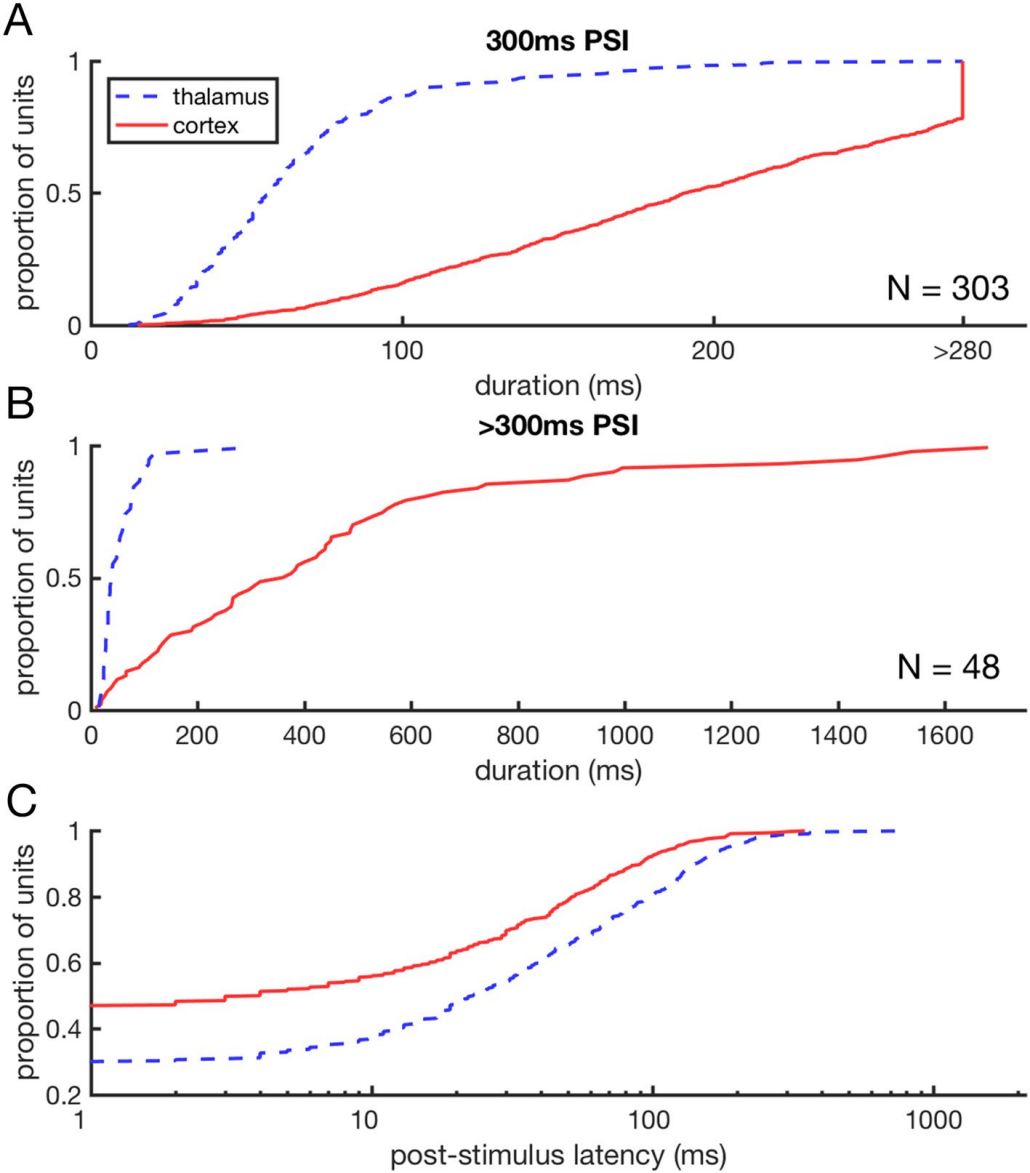
Post-stimulus activity in auditory thalamus. (**A**) Cumulative distribution of longest duration post-stimulus activity for thalamic units (broken line) presented with 200 ms stimuli followed by a 300 ms post-stimulus interval. Ns refer to thalamic units. The cumulative distribution of cortical response durations is shown for comparison (solid line). (**B**) Cumulative distribution of longest duration post-stimulus activity for thalamic units presented with stimuli that were followed by a PSI > 300 ms. Again, the cumulative distribution of cortical response durations is shown for comparison (solid line). (**C**) Cumulative distribution of post-stimulus activity latency in thalamus (broken line) and cortex (solid line).

Single units (N = 379) were also recorded from the auditory thalamus of 3 passively listening marmosets (Fig. 7A). The criteria for inclusion in further analysis was met by N = 361 units (see “Methods”). When presented with stimuli lasting 200 ms followed by a 300 ms PSI (N units = 303), thalamic post-stimulus activity was significantly shorter (median = 57 ms) than the post-stimulus activity reported above in cortex (N units = 805, median = 191 ms) (rank-sum test, p < 0.001) (Fig. 7A). Thalamic post-stimulus activity was also significantly shorter (N units = 48, median = 38.5 ms) than cortical activity (N units = 237, median = 196 ms) for units presented with stimuli followed by a PSI of > 300 ms (rank-sum test, p < 0.001) (Fig. 7B). The latency of post-stimulus activity, the time following stimulus termination before firing became significantly elevated, was significantly shorter in cortex (N units = 467, latency = 4 ms) than in thalamus (N units = 351, latency = 23 ms) (rank-sum test, p < 0.001) (Fig. 7C).

**Figure 8:**
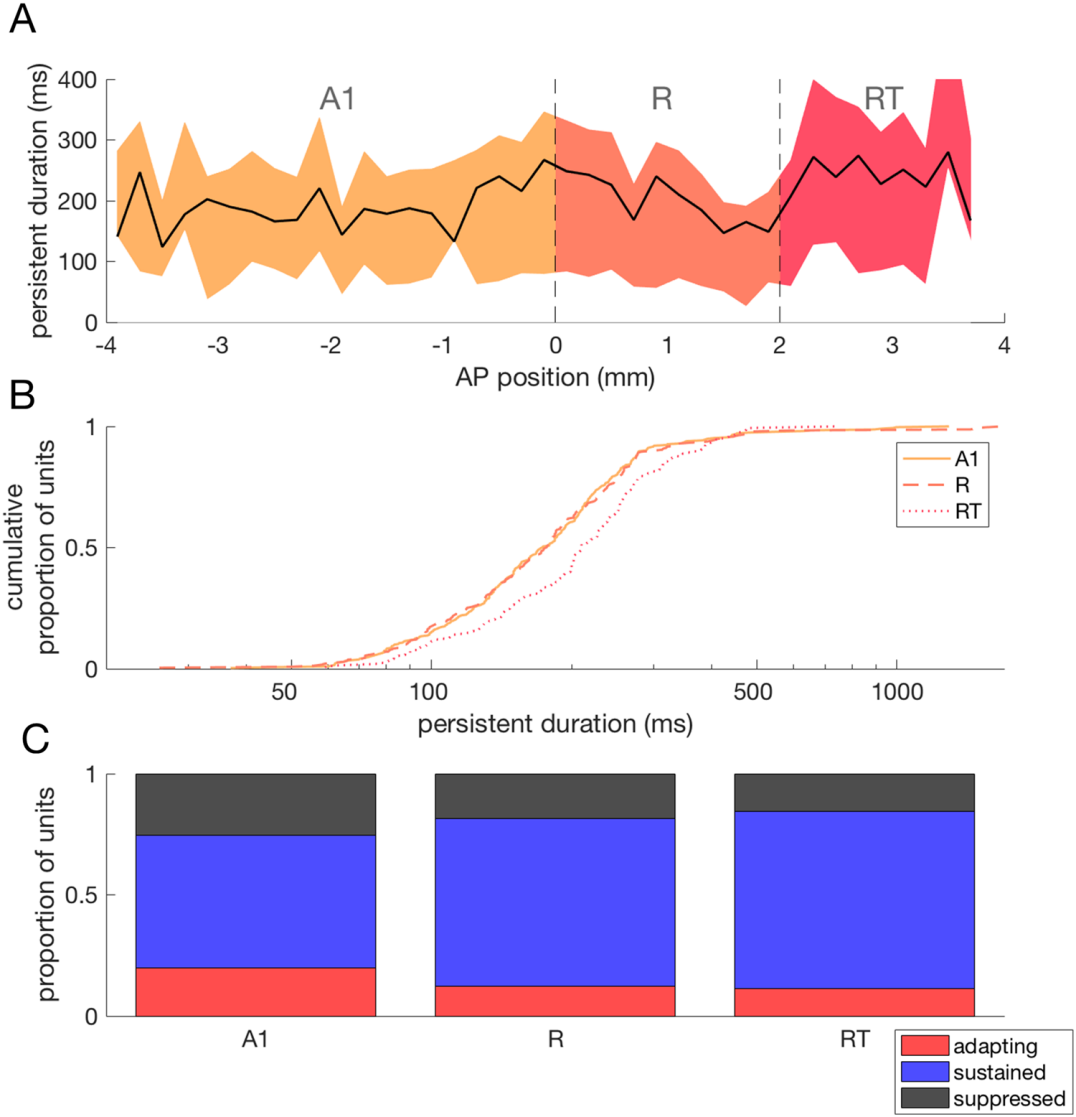
Variation in post-stimulus activity duration across auditory cortical fields. (**A**) Median (solid line) and inter-quartile ranges (coloured areas) of post-stimulus activity across the anterior–posterior extent of auditory cortex. Boundaries of cortical fields A1, R and RT are indicated with dashed lines. Data shown is combined from two subjects where recordings were made across the entire anterior–posterior extent of auditory cortex. (**B**) Cumulative distributions of post-stimulus activity across cortical fields showing a significant increase in duration in more anterior fields, R and RT. (**C**) Proportions of response types observed in different cortical fields, showing a trend towards a greater proportion of sustained responses in more anterior fields.

We next investigated whether variation in the duration of post-stimulus activity exists between cortical fields. Single units were recorded in areas A1, R and RT in core auditory cortex of two subjects, with units in each field being exposed to all stimulus types (Fig. 8A). In order to compare durations across fields it was necessary to only include units for which the maximum duration of post-stimulus activity could be accurately estimated, this is, where the duration of activity did not exceed the post-stimulus interval. The duration of post-stimulus activity showed significant variation across cortical fields (Kruskall–Wallis test, H(2) = 15.11, p < 0.001) (Fig. 8B). Post-hoc multiple comparisons of mean ranks indicated that post-stimulus activity of significantly longer duration in RT compared to A1 and R (median durations, A1 = 193.5 ms, R = 192 ms, RT = 233 ms; A1 vs R, p = 0.91; A1 vs RT, p < 0.001; R vs RT, p = 0.004). The pattern of longest duration responses being found in RT also held across individual subjects (Subject 1: Kruskall–Wallis test, H(2) = 12, p = 0.003; median durations, A1 = 159 ms, R = 175 ms, RT = 230.5 ms; A1 vs R, p = 0.82; A1 vs RT, p = 0.002; R vs RT, p = 0.017; Subject 2: Kruskall–Wallis test, H(2) = 4.45, p = 0.11; median durations, A1 = 172.5 ms, R = 177 ms, RT = 208 ms; A1 vs R, p = 0.996; A1 vs RT, p = 0.11; R vs RT, p = 0.16). Finally, we investigated whether the proportion of units showing each of the three within-stimulus response profiles (adapting, sustained, suppressed) varied across cortical fields. In A1 the majority of units that showed post-stimulus activity showed sustained responses in the within-stimulus period (54.95%; Fig. 8C). The proportion of units showing sustained responses was even greater in R (69.26%) and greater still in RT (73.14%). This indicates that response profiles preceding post-stimulus activity become more sustained in the more anterior auditory cortical fields.

## Discussion

Here we report the presence of post-stimulatory activity in a population of auditory cortical neurons in the absence of a behavioural task. We observed that, in the awake marmoset auditory cortex, these responses could last hundreds of milliseconds and in some cases over a second. Post-stimulatory activity has previously been observed in subcortical structures in non-primate species. In the dorsal cochlear nucleus of the cat pause-build units have been found to show modulation of spontaneous activity on the order of hundreds of milliseconds following the termination of auditory stimuli^42–44^. In the inferior colliculus of the anaesthetised mouse long-duration stimuli lasting over 30 s have been found to produce sound-evoked after discharges lasting several minutes^45^. The inheritance of post-stimulus activity from subcortical structures does not appear to account for the activity that we observed in cortex as we found post-stimulus activity to be of shorter duration in auditory thalamus than in cortex. Post-stimulus activity was also found to have a longer latency following stimulus offset in thalamus compared to cortex. This indicates that units displaying such activity in thalamus may simply inherit this activity via cortical feedback projections. We also observed that the duration of this activity increased in RT, a secondary cortical area. These findings indicate that the circuit architecture required to generate these responses may be intrinsic to cortical networks.

Post-stimulus activity was observed following several different response profiles during sensory stimulation. As previously reported, units in auditory cortex are capable of showing adapting and sustained responses^46^. The evoked responses of units observed here fell along a continuum from adapting, through sustained to ramping. Post-stimulatory activity was observed following both adapting and sustained response profiles, as well as following suppression. Given the heterogeneity of the stimulus-related activity that preceded the post-stimulus firing, the possibility that a single mechanism is responsible for this activity appears unlikely. In the prefrontal cortex, the mechanistic basis of post-stimulatory activity has not been fully elucidated but three broad classes of mechanism have been proposed to account for this pattern of activity; intrinsic neuronal properties at the cellular level, synaptic dynamics at the network level and the effect of activity from subcortical afferents at the systems level^47^.

The post-stimulus activity observed in sustained units could be accounted for by slow NMDA receptor-mediated excitatory synaptic currents which can last for hundreds of milliseconds^48^. These currents have been found to have a twofold longer decay time in rat prefrontal cortex compared to primary visual cortex, in keeping with these slow currents playing a role in the generation of prefrontal post-stimulatory activit^49^. It is not known whether NMDA currents in the marmoset auditory cortex are slower than in other cortical areas however. Cortical networks have a highly recurrent architecture^50^ and reverberant activity in such a network has long been considered a candidate mechanism for the generation of post-stimulatory activity^51–57^. A recurrent mechanism of this kind would account for the temporal fluctuations in post-stimulus activity that were widely observed in these units. It has been suggested that hybrid mechanisms are most likely responsible for post-stimulatory activity throughout the brain^58^ and, in keeping with this, combining slow excitatory currents with recurrent activity has been found to be important for the generation and stabilisation of post-stimulatory activity in computational models of such activity^55,56^. Similarly, asynchronous excitatory activity mediated by AMPA receptors has been found to be important for the maintenance of post-stimulatory reverberant activity in hippocampal cultures^59^. Sustained responses in auditory cortex that persist following stimulus offset and post-stimulatory activity in prefrontal cortex may therefore both be implemented by a hybrid of excitatory mechanisms. In keeping with an excitatory mechanism, the duration of post-stimulus activity following sustained responses was longer than for the other responses types. Furthermore, for sustained responses the peak firing rate in the within stimulus period predicted the duration of post-stimulus activity, indicating a positive relationship between excitatory drive during sensory stimulation and the duration of post-stimulatory activity observed. Future experiments could systematically vary sound duration in order to separate the mechanistic contribution of onset vs offset response dynamics to the generation of these responses.

Purely excitatory mechanisms do not seem capable of accounting for the full response profiles of adapting and suppressed response types however. Inhibition is known to play a role in the generation of adapting onset responses^60^ and thus may be responsible for the observed alteration in post-stimulus tuning observed for this class of responses. Greater adaptation of inhibitory synaptic input has been observed during sensory stimulation in neurons of the rat somatosensory cortex^61^. Such a pattern of adaptation could lead to excitation dominating during the postsynaptic period, providing a mechanism for post-stimulus firing in these units. Rebound calcium burst activity lasting hundred of milliseconds has been observed following sensory stimulation in the auditory thalamus^62^ and may contribute to the emergence of the post-stimulus activity observed in this area. This mechanism may also contribute to the generation of post-stimulus activity in adapting and suppressed cortical units as rebound activity is produced following inhibitory activity^63^. We also observed units that showed dramatic suppression in response to auditory stimuli, followed by the longest duration post-stimulus activity that we observed. This dramatic suppression is in keeping with a role for inhibition in generating this post-stimulus activity and this class of responses may share a common mechanism with the adapting response type. An imbalance in balance of excitation and inhibition during the post-stimulus period in favour of excitation may therefore represent a common mechanism for all three response types observed here.

The function of post-stimulus activity has been studied in a variety of behavioural contexts but the data presented here indicate that this activity may also play a role during passive listening. Echoic memory is a form of short-term sensory memory that differs from working memory in that it is posited to be active under all behavioral conditions, including passive listening^64^. The content of echoic memory is generally stored on the order of seconds^65–67^, a timescale that fits with the duration of post-stimulus activity observed here. An alternate possibility is that the activity reported here reflects the recruitment of circuits that exist in order to retain information in working memory and provide no function during passive listening. A functional role for this activity appears plausible, however, given the requirement of temporal integration in auditory perception in all species, from insects^68^ to humans^69^. Beyond the auditory domain, multi-sensory integration requires combining sensory information from multiple cortical areas with different latencies and temporal dynamics^70^. Such post-stimulatory activity may provide a substrate for integrating disparate sensory signals into a coherent percept of an object. Investigating the function of activity observed in the absence of a behavioural task is particularly challenging however. Experiments addressing the function of this activity could exploit behavioural measures that provide an implicit measure of the processing of sensory information in the absence of a task, such as fear-conditioned freezing^71^. Combining this approach with temporally precise suppression of this activity through optogenetic methods, it may be possible to investigate the potential role of this activity in echoic memory and temporal integration. Developing an approach that will enable the investigation of the potential roles of post-stimulatory activity during passive listening will be crucial in elucidating the functional properties of such activity.

## Methods

The data analysed in this study were obtained from several previous experiments conducted in the Laboratory of Auditory Neurophysiology at Johns Hopkins University School of Medicine in the laboratory of Prof. Xiaoqin Wang^37–41^. All experimental procedures were approved by the Johns Hopkins University Animal Use and Care Committee and were carried out in accordance with relevant guidelines and regulations. The methods to record single-unit activity in awake marmosets were previously described^61^ and are briefly summarised below.

Single-unit recordings from auditory cortex and thalamus were conducted in 7 awake, passively listening marmosets sitting on a semi-restraint device with their head immobilized, within a double-walled soundproof chamber (Industrial Acoustics). The inside wall of the chamber was covered by 3-inch acoustic absorption foam (Sonex). For auditory cortical recordings high-impedance tungsten microelectrodes (3–5 M, A-M Systems) were inserted perpendicular to the cortical surface. Electrodes were mounted on a micromanipulator (Narishige) and advanced by a manual hydraulic microdrive (Trent Wells). Action potentials were detected on-line using a template-based spike sorter (Multi-Spike Detector; Alpha Omega Engineering) and continuously monitored by the experimenter while data recording progressed. Typically 5–15 electrode penetrations were made within a miniature recording hole (diameter 1 mm) over the course of several days, after which the hole was sealed with dental cement and another hole opened for new electrode penetrations. Neurons were recorded from all cortical layers, but most commonly from supragranular layers.

### Generation of acoustic stimuli

Acoustic stimuli were generated digitally and delivered by a free-field loudspeaker located one meter directly in front of the animal. All sound stimuli were generated at a 100 kHz sampling rate and low-pass filtered at 50 kHz. The sound level of individual frequency components used in this study was no higher than 80 dB SPL. Frequency tuning curves and rate-level functions were generated using pure-tone stimuli of 200 ms in duration with post-stimulus intervals of 500 ms, and had a minimum of five repetitions each. Stimulus presentation order was fully randomised. Pure-tone stimuli intensity levels were generally 10–20 dB above threshold for neurons with monotonic rate-level functions, or at preferred levels for non-monotonic neurons. Broadband rectangular clicks or narrowband clicks made of brief pulses of white noise or a tone (at an integer multiple of the frequency) were used to generate click trains. Rectangular click trains had a width of 0.1 ms while narrowband clicks had each pulse convolved with a Gaussian envelope with a standard deviation of 0.1–0.4.

### Identification of cortical fields

Single units with significant neuronal discharges to narrowband stimuli, such as tones and band-pass noise, were used to generate cortical characteristic frequency maps. The characteristic frequency of each location on the map was determined by the median characteristic frequency of all electrode tracks within 0.25 mm. Electrode track characteristic frequencies were calculated by computing the median characteristic frequency of units within the track. The anterior–posterior position is reported relative to the boundary between A1 and R. Both the boundary between A1 and R as well as the boundary between R and RT were identified from the frequency reversal between the cochleotopic gradients of the two fields, as their maps are mirror-reversed^72–75^. Please see^38^ for more details.

### Quantification of post-stimulus activity duration

**Figure 9:**
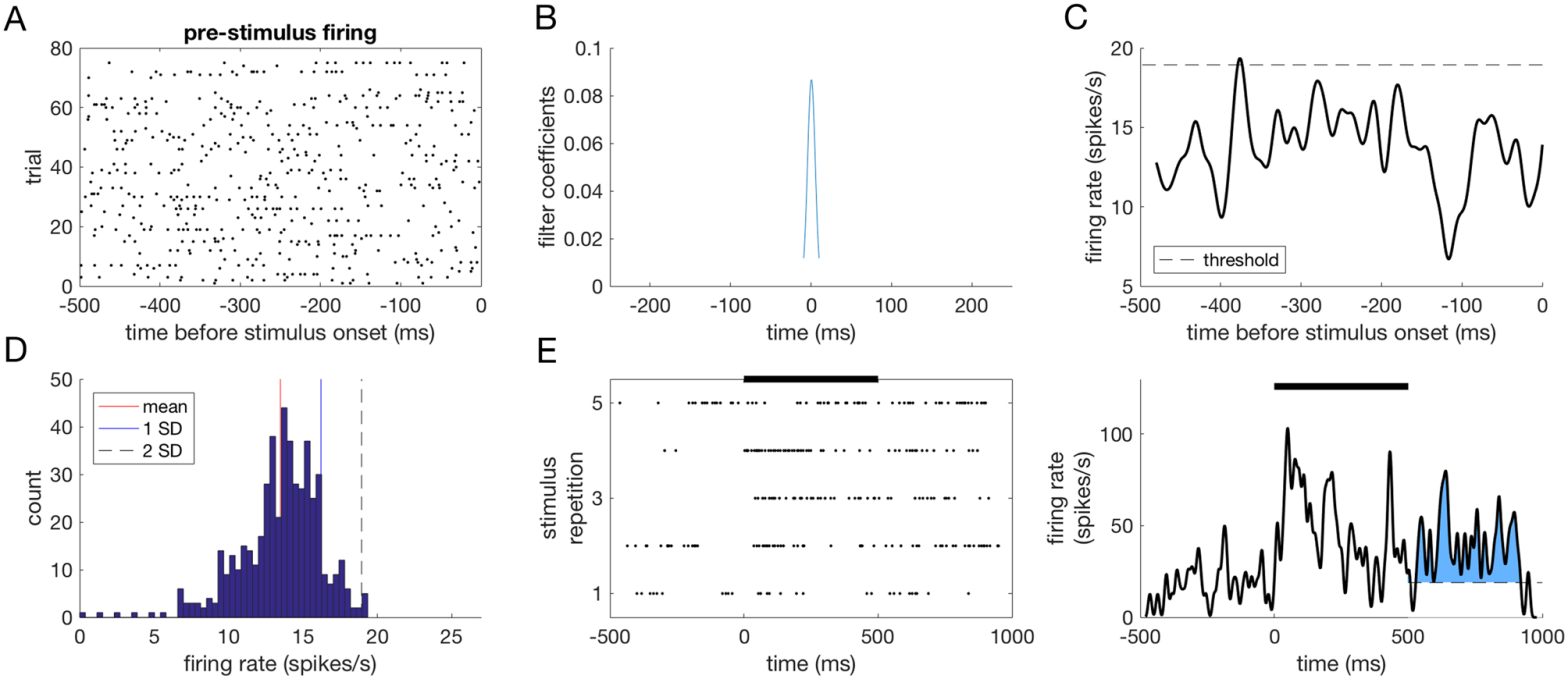
Quantification of post-stimulus activity duration. (**A**) Pre-stimulus firing for one neuron for all stimuli presented. (**B**) Bi-directional Gaussian filter (σ = 5 ms, total bandwidth = 20 ms), used to calculate instantaneous firing rate during rate. (**C**) Pre-stimulus instantaneous firing rate, calculated by convolving the spike trains in A with the filter in B and normalizing by the number of stimulus repetitions. The dashed line is the threshold for significantly elevated firing, positioned at 2 standard deviations (SD) above mean firing rate. (**D**) The distribution of firing rate values in the trace in (**C**), showing the mean firing rate (red line), 1 SD above the mean (blue line) and 2 SD above the mean (broken black line). The latter is equivalent to the threshold for significantly elevated firing, as shown in (**C**). (**E**) Left panel: Firing for the same neuron on a subset of trials, corresponding to five repetitions of a single stimulus. The black bar indicates the duration of the 500 ms bandpass noise stimulus. Right panel: Instantaneous firing rate calculated by convolving the spike train in (**E**) with the filter in (**B**). The broken black line indicates the threshold shown in (**C**) and (**D**), as calculated from pre-stimulus firing. The area shaded in blue above the threshold indicates the duration of significantly elevated post-stimulatory activity.

Quantification of post-stimulus activity was based on significant difference from baseline firing. Baseline firing rate was calculated from a pre-stimulus interval that was separate from the post-stimulus interval of the preceding stimulus (Fig. 9A). A wide range of pre-stimulus interval, post-stimulus interval, stimulus duration and stimulus type combinations were used resulting in 82 different stimulation conditions. Several subsets with consistent durations were used in our analysis. Stimuli that had a post-stimulus interval of 300 ms had a pre-stimulus interval of 200 ms, creating an inter-tone interval of 500 ms. Stimuli that had a post-stimulus interval of 500 ms had a pre-stimulus interval of 500 ms, creating an inter-tone interval of 1,000 ms. For stimuli with a post-stimulus interval of > 500 ms, a range of pre-stimulus intervals from 200 to 8,000 ms were used that varied with the duration of the particular stimulus. Units were required to have a baseline firing rate, calculated across all pre-stimulus periods, of > 1 spike per second and to have been presented with 5 or more repetitions of each stimulus to be included in all further analysis. This criterion was met by 1,188 units of 1,557. The duration of post-stimulus activity was quantified by convolving spike trains for a given stimulus with a bi-directional Gaussian filter (σ = 5 ms, total bandwidth = 20 ms) in order to estimate the mean instantaneous firing rate before, during and after the presentation of the stimulus (Fig. 9B). The same method was used in order to generate a mean firing rate trace for the pre-stimulus period, combining the data from all pre-stimulus periods for the unit (Fig. 9C). The mean and standard deviations of baseline firing were calculated from this trace (Fig. 9D). Significant post-stimulus activity was required to be > 2 standard deviations over mean baseline firing.

Significant post-stimulus activity was defined as periods of firing that crossed the threshold (Fig. 9E). Firing during this period was required to be over the threshold on at least half of the trials, in order to avoid large single trial events being mistaken for reliable post-stimulus activity. The observed post-stimulus activity could be highly variable over time. Therefore, momentary drops below this threshold (< 20 ms) were not considered in our estimate of post-stimulus activity duration. In order to control for chance fluctuations in firing rates, the duration of elevated firing during spontaneous activity during the pre-stimulus period was also calculated. The duration of post-stimulus activity was required to be above the range of values that were observed by chance in the pre-stimulus period for each neuron.

### Ethical approval

All experimental procedures were carried out at Johns Hopkins University in AAALAC approved facilities and were approved by the Johns Hopkins University Animal Use and Care Committee (IACUC). All methods were carried out in accordance with relevant guidelines and regulations.
